# Laparoscopic Orthotopic Kidney Transplantation in Swine: A Novel Vascular Prop Device for Venous Anastomoses

**DOI:** 10.3389/fsurg.2021.708449

**Published:** 2021-08-26

**Authors:** Peng Zhang, Xiu-wu Han, Xin Zhang, Xu-hui Zhu, Tao Li, Yan-sheng Li, Yuan-hao Chen, Gao Li, Long-xi Han, Rong-jie Zhang

**Affiliations:** Department of Urology, Beijing Chao-Yang Hospital, Capital Medical University, Beijing, China

**Keywords:** laparoscopy, orthotopic kidney transplantation, venous anastomosis, prop device, technique

## Abstract

**Objective:** To investigate the safety and efficacy of a vascular prop device for laparoscopic orthotopic kidney transplantation (LOKT) in swine.

**Material and Methods:** Twenty swine were randomly divided into two groups. A vascular prop device was used in the observation (VP) group, and the vein beltization technique was used in the control (VB) group. The right kidney, as a donor graft, was laparoscopically transplanted to the location of the left kidney after a left nephrectomy. Data on the operative time, venous anastomotic time, vein stenosis, etc., and the survival of the swine in the two groups were recorded.

**Results:** The mean transplant operative time, the mean cold ischemia time, and the venous anastomotic times in the VP group were significantly shorter than those in the VB group. Seven swine in the VP group and three swine in the VB group survived for 7 days. Autopsy results showed the occurrence of one artery stenosis and one vein stenosis in the VP group and one artery stenosis and five vein stenoses in the VB group. The median survival time was 6.25 days for the swine in the VP group and 4.40 days for those in the VB group.

**Conclusions:** The vascular prop device is safe and feasible for LOKT in swine and may accelerate venous anastomosis and ensure the quality of venous anastomotic stoma.

## Background

Heterotopic kidney transplantation has been used for more than a century, and it has proven to be a successful technique for kidney transplantation. However, this technique has still not been perfected. Based on observations of a large number of kidney transplant recipients and our clinical experience, we can infer that laparoscopic orthotopic kidney transplantation (LOKT) is probably the most logical type of kidney transplant from an anatomical, physiological, and psychological point of view. Given the anatomical position of the kidney, open surgery for orthotopic transplantation is both traumatic and difficult. Clinically, it is only used for patients whose iliac vessels are not suitable for heterotopic kidney transplantation (1–5). It is hoped that the development of laparoscopic techniques may provide an opportunity for improved orthotopic kidney transplantation. The application of LOKT has been reported in the literature and shows promise (6–10), and one case of orthotopic robot-assisted kidney transplantation has recently been reported (11). LOKT requires not only reliable intraoperative cooling measures to ensure the hypothermia and good function of the transplanted kidney, but also reliable vascular anastomosis techniques to ensure the anastomosis quality of the transplanted kidney vascular and good blood supply. During our previous study, we found that the most difficult procedure associated with LOKT was renal-vein anastomosis (6, 7, 12). Based on the experience of a large number of animal experiments, we designed a novel vascular prop device that can help to open the veins of the kidney, facilitate vascular anastomosis, greatly improve the speed and quality of vein anastomosis, and improve the success rate of LOKT. In this study, swine, a large animal, were used to conduct a controlled study to verify the effectiveness and safety of this technique.

## Materials and Methods

### Study Design

The study was approved by the animal ethics committee of Beijing Chao-Yang Hospital. Swine (Ba-Ma mini pigs) were raised in Beijing Tonghe Litai Biotechnology Co., Ltd. With a 12 h light/dark cycle, a constant temperature of 20–25°C, and 40–70% relative humidity. Single cage (90 × 100 × 100 cm) feeding mode was adopted, one swine per cage, and the feeding cage was made of standardized stainless steel. The piggery is cleaned up excrement daily, rinsed once a week and thoroughly disinfected once a month. The raising environment is enriched by means of the iron chain hanging on the cage wall, the floor covered with hay mat, adding toys and other measures. Environmental imaging of animals is <60 decibels. Provide 0.5–1 h of outdoor activity per day. Keepers are required to work closely with each animal and establish a trusting relationship with the pigs through smell, sound, feeding and touch. Swine were fed the standardized swine feed produced by Beijing Ke'ao Cooperation Co., Ltd., each pig was fed 3% of its body weight twice a day and allowed free access water. All swine were quarantined and vaccinated regularly. Two days before the experiment, the swine (40 ± 5 kg) were transported to the animal experimental laboratory. All swine were fasted for 12 h before surgery.

Animal anesthesia were similar to those of our previous study (12). Anesthesia was induced by intramuscular injection of Xylazine Hydrochloride (at dose of 40 mg/kg) and Zoletil^™^ 50 (at dose of 5 mg/kg). During the whole operation, isoflurane inhalation anesthesia was performed with a respiratory anesthesia machine. The tidal volume (mL) was set as 10 times of the animal's body weight value (Kg), and the respiratory rate was set as 15 times/min. The anesthesia was maintained with 1% isoflurane, which changed from spontaneous breathing to passive breathing. During the operation, anesthetic monitor was used for routine vital signs (heart rate, oxygen saturation, non-invasive blood pressure, and body temperature) monitoring.

Twenty swine were randomly divided into two groups. LOKT was performed on both groups. The renal-vein prop device was employed in the observation (VP) group, and the renal-vein beltization technique, as reported in our previous study (12), was employed in the control (VB) group. First, a right nephrectomy was performed. As the donor kidney, the right kidney was immediately perfused and stored in perfusion fluids [hypertonic citrate adenine solution, developed by Chang zheng Hospital Affiliated to the Second Military Medical University (Shanghai, China)] at 0–4°C. The right kidney was prepared either for vascular prop-device usage with the swine assigned to the VP group or for the vein beltization technique for the swine assigned to the VB group. After the left nephrectomy was performed, the right kidney was laparoscopically transplanted to the location of the left kidney. A homemade net-restrictive double-layered plastic jacket (Chinese patent no.: ZL2018 2 0687164. X) was used in both groups for graft cooling, as in previous studies (13, 14). The operative time, venous anastomotic time, vein stenosis, blood loss, kidney-graft function, and survival of the swine in the two groups were recorded. After 1 week, the renal graft was removed from the surviving swine under anesthesia for inspection and pathologic examination.

### Design of the Vascular Prop Device

**Figure 1:**
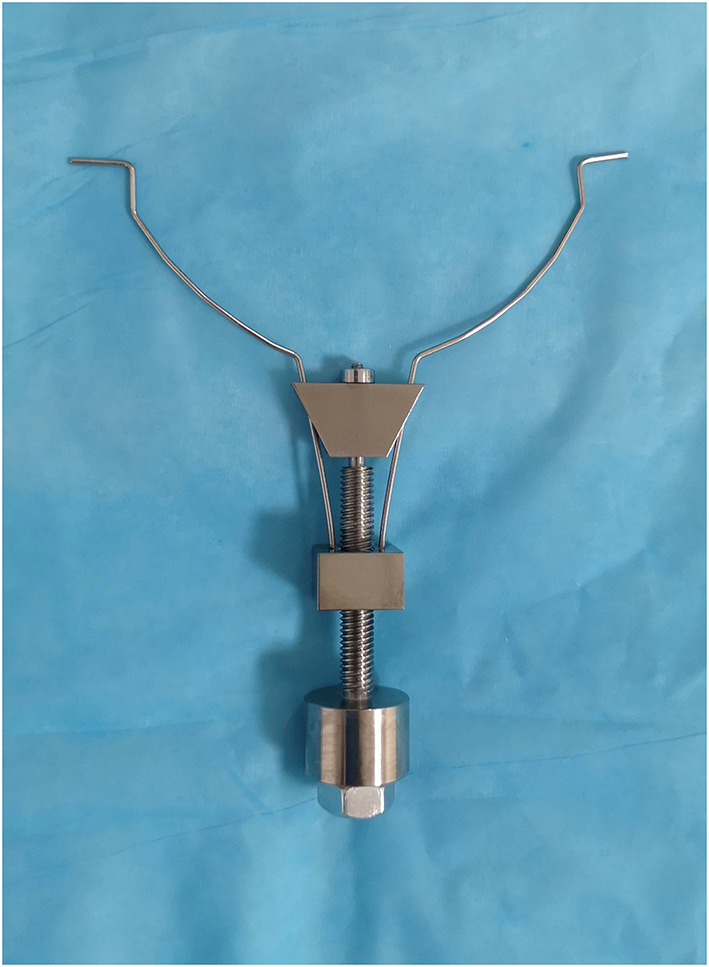
The vascular prop device model.

The vascular prop device is composed of a metal nut plate, adjusting plate, and bolt structure. A small hole is located in the middle of the nut plate, which is the nut structure. The two springs fixed to the small metal block of the nut plate constitute a symmetrical structure, and the ends of the two springs are hooked outward. A small hole is located in the middle of the regulating plate, and two thin holes are located at either end of the plate. The regulating plate is held by a grooved structure at the top of the bolt, and a spring wire passes through two symmetrical holes. The distance between the two spring ends is adjusted by moving the regulating plate up and down by rotating it in different directions using the bolt–nut relationship. This vascular support device model is shown in Figure 1.

### Nephrectomy Surgery

The surgical procedures were performed under sterile conditions. A total of 3,000 IU of IV heparin was given for the prophylaxis of blood clots. A 10- to 12-cm midline incision was made for both side nephrectomies in the supine position. Nephrectomies were performed from the retroperitoneum. For the right nephrectomy, the renal artery and vein were transected in position up to the aorta and the vena cava. For the left nephrectomy, the renal vascular trunk was left as long as possible by dissecting the vessels up to the hilum. The left renal artery and vein were temporarily clipped using a bulldog and titanium clip, respectively.

### Autotransplantation Surgery

**Figure 2:**
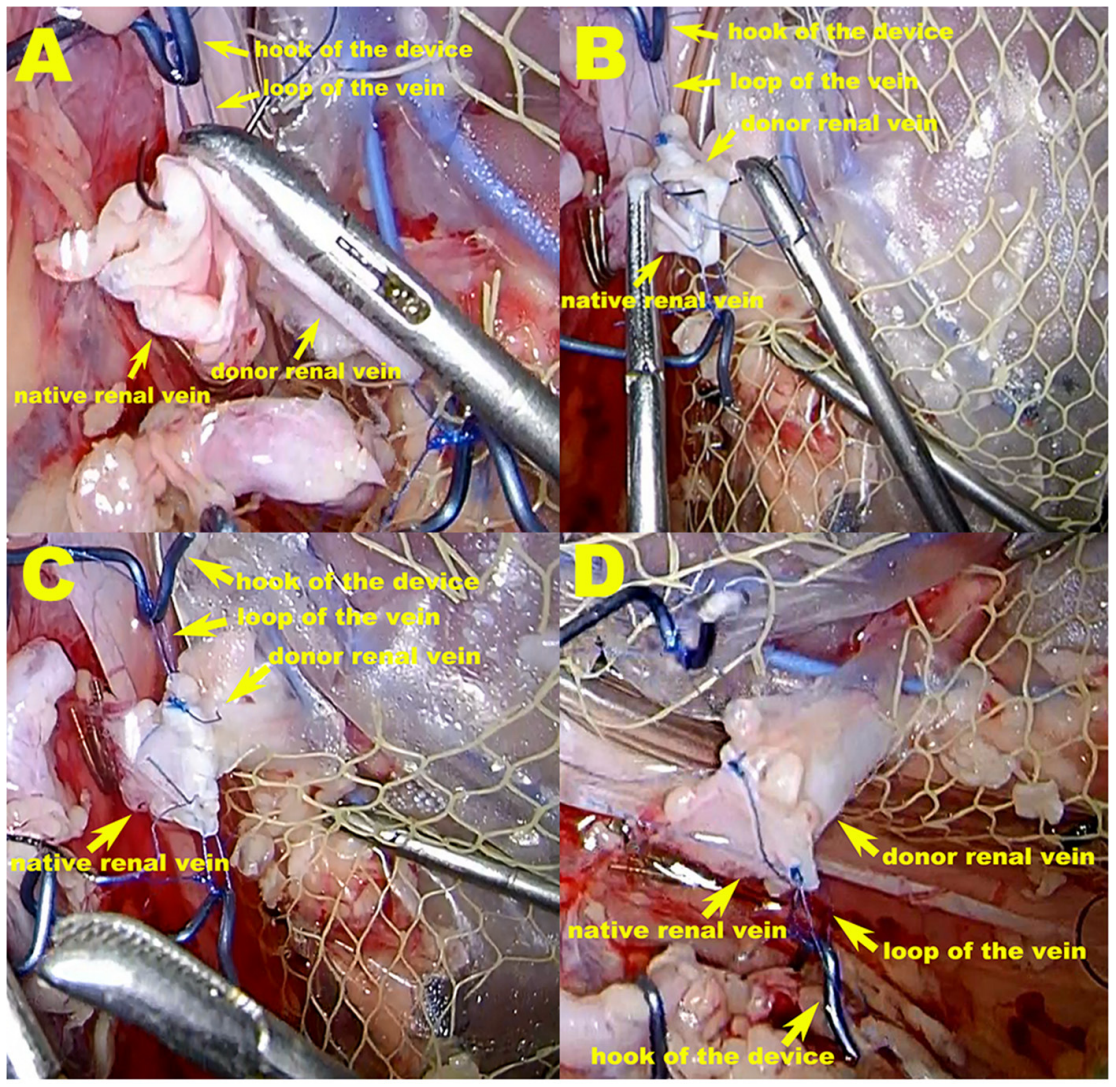
The vascular prop device in operation in the VP group. **(A,B)** During the operation, the hook of the prop device holds the Prolene suture material loop to prop open the donor vein, thereby tightening the renal vessels at the maximum diameter. After vein-to-vein fixation at the upper and lower sites of the donor and native vein, respectively, the native vein is also supported and pulled out, which facilitates vein anastomosis. **(C,D)** Venous anastomosis completed. The purse-string effect at the vein anastomotic stoma, which can be seen in the VB group, is not possible because excessive suture contraction is avoided.

In the VP group, the vascular prop device was used for renal-vein anastomosis. The right kidney was prepared for this device by suturing and tying a knot to form a small loop using Prolene suture material at the upper and lower edge of the vein, respectively. During the operation, the hook on the prop device held the Prolene suture material loop to prop open the donor vein. After vein-to-vein fixation at the upper and lower sites of the donor and native veins, respectively, the native vein was also supported and pulled out, thereby facilitating vein anastomosis (Figure 2).

**Figure 3:**
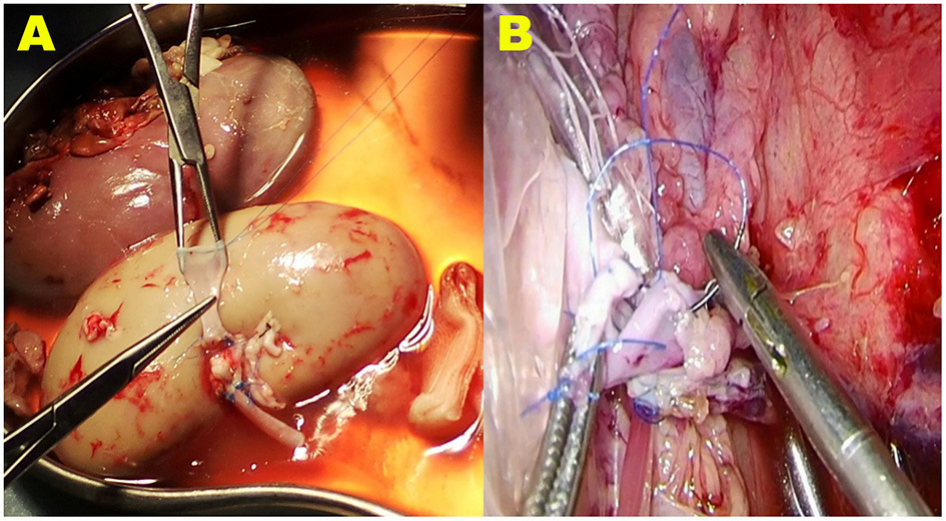
The vein beltization technique used for vein anastomosis in the VB group. **(A)** The broken end of the donor renal vein is circumferentially sutured with a 5-0 Prolene suture material at a distance of 2.0 cm from the edge. When the knot is tied, the vein is braced with forceps. This circumferential circle of Prolene sutures can widen the vein to its maximal size. **(B)** During the operation, when tightening the suture material and tying the last knot to finish the anastomosis, the ends of the Prolene suture material are carefully pulled; however, avoiding a purse-string effect at the anastomosis stoma of the vein is difficult.

In the control group, the renal-vein beltization technique was used for renal-vein anastomosis. For this technique, the broken end of the donor renal vein was circumferentially sutured with a 5-0 Prolene suture material (Ethicon, Somerville, NJ, United States) at a distance of 2.0 cm from the edge, with a 2-mm interval between each stitch, and then a knot was tied. After tying the knot, the vein was braced with forceps. This circumferential circle of Prolene sutures can widen the vein to its maximal size (12) (Figure 3A).

A homemade net-restrictive plastic jacket, as described in our previous study, was used to maintain hypothermia in both groups. Briefly, the plastic jacket is composed of a sealed plastic bag with two silicone tubes and a small plastic mesh bag. The bottom of the plastic bag is dented to form a new cavity, and the donor kidney is placed in the cavity. The opening of the cavity is then closed with titanium clips, leaving a gap in the middle for the vessels to pass through. The plastic bag (including the kidney) is wrapped and trussed tightly in the mesh bag, which is then cut open to form a small hole at the renal hilum to expose the renal artery and vein anastomosis. This allows nearly the entire kidney to be surrounded by the circulating perfusion of saline ice water at 0–4°C to achieve a cooling effect (13, 14) (**Figures 2, 3B**).

After the left nephrectomy, the animal was repositioned to lie on its right side. The cooling device with the graft was positioned in the left renal location through the midline incision. The wound was then temporarily closed for LOKT. The right kidney graft was implanted in the location of the left kidney with the end-to-end technique used to complete the anastomosis of the artery and vein with 5-0 Prolene sutures. Real-time monitoring of the temperature of the graft kidney was performed by a multichannel temperature tester (JK808, Changzhou Jin Ai Lian Electronic Technology Co., Ltd, Changzhou, China) during renal artery and vein anastomoses. Following the release of the venous bulldog clip and then the arterial bulldog clip, reperfusion was performed. After a 10 French catheter was inserted into the ureter, cutaneous ureterostomy was carried out for the continuous observation of urine output of the renal graft. Finally, the middle incision was closed.

After surgery, the swine were actively cared for and kept warm until they were fully awake and transferred to cages, and had free access to food and water. Intravenous analgesia were administered PRN. Intravenous hydration was maintained by infusion of 0.9% normal saline (10 ml/kg/h) until day 1 after the surgery. In addition, 1,500 IU heparin per day was administered subcutaneously. Penicillin sodium for injection (800,000 units per animal) was injected intramuscularly for anti-infection every day for 7 days. The degree of pain was evaluated according to the clinical symptoms of the pigs after operation and the USDA pain levels, and corresponding analgesic treatment was given. Subcutaneous injection of Carprofen (at dose of 0.5–4.0 mg/kg) was given every day for 3 days. In addition, subcutaneous injections of meloxicam (at dose of 0.1–0.3 mg/kg) or buprenorphine (at dose of 0.01–0.05 mg/kg) were administered as appropriate, depending on the pain level of the animal. Iodophor solution was used to disinfect the surgical wound every day. Urine output was recorded, and blood samples were taken daily. B-ultrasonography was used to examine the vessel anastomoses and graft blood supply and to identify any surgical complications. On post-transplantation day 7, a second laparotomy was performed on the surviving swine under anesthesia, and the transplanted kidney graft was examined and removed. The swine were then euthanized at the end of surgery (intravenous injection of potassium chloride solution). Graft histopathology was performed under light microscopy.

### Statistical Analysis

Data are presented as the mean ± standard deviation. Statistical analyses were performed with *t*-tests using SPSS 17.0 software. The survival rate was calculated using the Kaplan–Meier method, and the log-rank test was used to compare the survival curves. A significance level of *P* < 0.05 was considered sufficient in all experiments.

## Results

**Figure 4:**
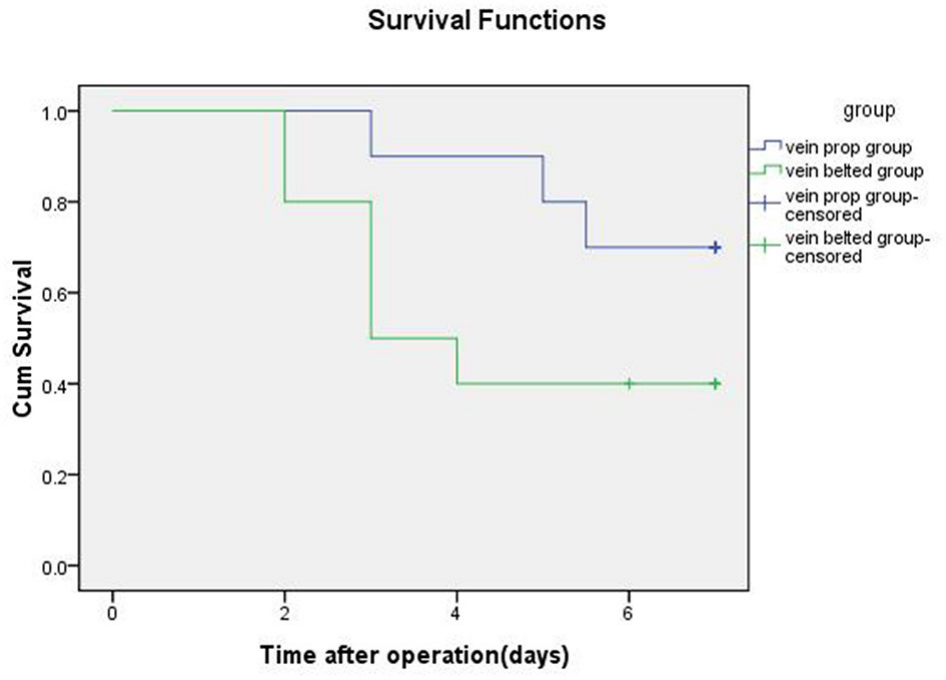
Kaplan-Meier survival curve of the two groups receiving renal allografts.

All LOKT procedures were completed in both groups. There were no differences between the two groups in terms of the warm ischemia time, artery anastomotic time, surface temperature of the graft, and artery stenosis. However, the mean transplant operative time, the mean cold ischemia time, and the venous anastomotic times in the VP group were significantly shorter than those in the VB group. After reperfusion of the blood, a satisfactory blood supply was observed in eight grafts based on ultrasonic examination in the VP group and five grafts in the VB group. Four of the 10 grafts showed diuresis during the operation in the VP group, and one graft showed diuresis in the VB group. Seven swine survived for 7 days with mean serum creatinine levels of 230 ± 32 μmol/L in the VP group, and three swine survived for 7 days with mean serum creatinine levels of 410 ± 21 μmol/L in the VB group. Autopsies verified one artery stenosis and one vein stenosis in the VP group and one artery stenosis and five vein stenoses in the VB group. Thrombosis was seen in the stenosis of the vessel. A histopathologic examination of the autografts demonstrated near-normal renal architecture in four of the seven swine that survived for 7 days in the VP group and only one of the three that survived in the VB group. The autografts also showed that six swine had acute tubular necrosis in the VB group and three in the VP group. The median survival time was 6.25 days for the swine in the VP group and 4.40 days in the VB group. The detailed data and results from the two groups are shown in Table 1 and Figure 4, respectively.

**Table 1:** The observational data from the two groups.

**Variables**	**VP**	**VB**	***p*-value**
Number (*n*)	10	10	
Operation time (h)	4.1 ± 0.2	5.3 ± 1.3	0
Warm ischaemia time (min)	7.0 ± 0.7	6.8 ± 0.9	0.879
Cold ischaemia time (h)	3.5 ± 0.1	5.1 ± 0.3	0.001
Vein anastomotic time (min)	45 ± 18	68 ± 8.0	0
Artery anastomotic time (min)	25 ± 19	30 ± 12	0.645
Mean blood loss (mL)	55 ± 12	75 ± 10	0.067
Surface temperature of graft (°C)			0.564
Upside	5.7 ± 2.3	5.5 ± 1.4	0.001
Underside	6.2 ± 1.3	5.2 ± 1.3	0.01
Artery stenosis (*n*)	1	1	
Vein stenosis (*n*)	1	5	
Wound infection (*n*)	1	1	
Urine output at 7 day (ml)	1,606 ± 30	850 ± 235	0.076
Scr at 7 day (umol/dl)	230 ± 32	410 ± 21	0.002
BUN at 7 day (mg/dl)	20.8 ± 2.2	25.6 ± 3.4	0.042
ATN (*n*)	3	6	
Mean survival time (d)	6.25	4.4	0.046

## Discussion

This new operation method for LOKT conforms to human anatomical, physiological, and psychological requirements. Theoretically, LOKT (including robot-assisted LOKT) should represent the best technique for kidney transplantation. Regardless of whether LOKT can be safely applied and widely performed in clinical practice, we have addressed the intraoperative organ cooling problem, and we have made a reliable vascular anastomosis. Great efforts should be made to ensure that there is no stenosis, torsion, stricture, or leakage resulting from the vascular anastomosis, and postoperative complications should be avoided. These are prerequisites for the application of orthotopic kidney transplantation.

LOKT has also been attempted in a swine model in which laparoscopic instruments were used to complete open surgery-like vascular anastomosis. In terms of vascular-anastomosis technological innovations in laparoscopic transplantation, there are few reported in the literature. We have described the renal-vein beltization technique for LOKT in a swine model (12). This technique is helpful for venous anastomosis and promotes the quality of vessel reconstruction in LOKT, but a purse-string effect at the anastomosis is difficult to avoid. Therefore, a novel vascular prop device has been designed (patent application number: 202022174071.7). This device can pull out and brace the vessels when the vessels are anastomosed, which can facilitate the completion of reliable vascular anastomosis.

This vascular prop device has the following advantages: 1. it tightens the renal vessels at the maximum diameter to facilitate anastomosis; 2. when knotting, a purse-string effect at the vein anastomosis stoma, caused by excessive suture contraction, can be avoided; and 3. if the vessels are in good condition after anastomosis is complete, the vessels can be rotated over 90–180° to facilitate contralateral vascular suturing, completing an eversion suture anastomosis. In addition, this device can easily enter and exit the laparoscopic 10-mm trocar, and the bolts can be rotated using a laparoscopic approach to adjust the tension of the spring-wire hook to avoid tension that is too loose or too tight. The shortcomings of this study are as follows: the number of swine used in the study was small, the observation time could have been longer, and the prop device was made in house. This device should be refined; it will perform better if it is produced as a commercial product.

The key techniques mentioned above, such as renal graft cooling and vascular anastomosis, can also be extended to robot-assisted LOKT surgery. Therefore, it is worth further study and improvement.

## Conclusion

Our study shows that the vascular prop device is beneficial to swine venous anastomosis and could improve the quality of venous anastomotic stoma for LOKT. The device is safe and feasible for use in LOKT, and it can reduce the cold ischemia time and help to achieve better postoperative effects. The use of this vascular prop device for venous anastomoses in LOKT is recommended based on research performed in swine models.

## Data Availability Statement

The original contributions generated for the study are included in the article/supplementary material, further inquiries can be directed to the corresponding author.

## Ethics Statement

The animal study was reviewed and approved by the animal ethics committee of Beijing Chao-Yang Hospital.

## Author Contributions

PZ, GL, and XH conceived the idea and conceptualised the study. PZ and XZ collected the data. XZ and TL analysed the data. PZ, RZ, and YL drafted the manuscript. XH, LH, and YC reviewed the manuscript. All authors read and approved the final draft.

## Conflict of Interest

The authors declare that the research was conducted in the absence of any commercial or financial relationships that could be construed as a potential conflict of interest.

## Publisher's Note

All claims expressed in this article are solely those of the authors and do not necessarily represent those of their affiliated organizations, or those of the publisher, the editors and the reviewers. Any product that may be evaluated in this article, or claim that may be made by its manufacturer, is not guaranteed or endorsed by the publisher.
